# Supplementary material for: Decoding the genetic and chemical basis of sexual attractiveness in parasitic wasps
Source: eLife. 2023 Jul 11;12:e86182. doi: 10.7554/eLife.86182 (PMC10435230; doi:10.7554/eLife.86182)
Supplement: Supplementary file 1. — All primers were designed using the Primer-BLAST tool from the National Center for Biotechnology Information (NCBI). Indicated are the respective primer names, their sequences, and their usage in the experimental protocol. [file elife-86182-supp1.docx]

Supplementary File 1. **List of primers used in the present study.** All primers were designed using the Primer-BLAST tool from the National Center for Biotechnology Information (NCBI). Indicated are the respective primer names, their sequences, and their usage in the experimental protocol.

| **Primer name** | **Sequence** | | **Usage** | |
| --- | --- | --- | --- | --- |
| *fas5*_dsRNA_F | GATGCAAAGACCAACAAAGCC | | dsRNA synthesis for targeting *fas5* | |
| *fas5*_dsRNA_R | GCAAAGATTTCGCGATTCCTG | |  |  |
| *fas5*_dsRNA_T7_F | TAATACGACTCACTATAGGGGATGCAAAGACCAACAAAGCC | |  |  |
| *fas5*_dsRNA_T7_R | TAATACGACTCACTATAGGGGCAAAGATTTCGCGATTCCTG | |  |  |
| GFP_dsRNA_F | GTGACCACCTTGACCTACGG | | dsRNA synthesis for targeting GFP | |
| GFP_dsRNA_R | TCTCGTTGGGGTCTTTGCTC | |  |  |
| GFP_dsRNA_T7_F | TAATACGACTCACTATAGGGGTGACCACCTTGACCTACGG | |  |  |
| GFP_ dsRNA_T7_R | TAATACGACTCACTATAGGGTCTCGTTGGGGTCTTTGCTC | |  |  |
| *fas5*_qPCR_F | CTATGTTTGATGATATCAAGGCTGA | | quantitative (q) PCR for ***fas* geneexpression** | |
| *fas5*_qPCR_R | CATATGTATACTGGTCCCCGTAAG | |  |  |
| ***fas6*_qPCR_F** | | **GGGACCACACAAATCAAAATTGTA** | |  |
| ***fas6*_qPCR_R** | | **GTTTTCTCTCCTTCAATCACACTCA** | |  |
| ***fas1*_qPCR_F** | | **TGAGAAACACAAGCCTACCGA** | |  |
| ***fas1*_qPCR_R** | | **TTGACTGTCTTGACGTCCTTGA** | |  |
| ***fas2*_qPCR_F** | | **AATCAAACCTGCCTCGGGTG** | |  |
| ***fas2*_qPCR_R** | | **GCAGCAAGGCTTCTCCCATT** | |  |
| ***fas3*_qPCR_F** | | **AGTTCTCCCGTCTCGGTGTA** | |  |
| ***fas3*_qPCR_R** | | **GTGAACGAAGGTGGCGTAGA** | |  |
| ***fas4*_qPCR_F** | | **ACTGCTTTGGAGCTTGCGTA** | |  |
| ***fas4*_qPCR_R** | | **TCCGCAGATAAAACACCTAAGGC** | |  |
| *Elf1a*_qPCR_F | GAGCCATCCACCAAAATGCC | | quantitative (q) PCR for *Elf1a* **expression ( housekeeping gene)** | |
| *Elf1a*_qPCR_R | CTTGTAGACGTCCTGGAGGG | |  |  |
