# Supplementary material for: Decoding the genetic and chemical basis of sexual attractiveness in parasitic wasps
Source: eLife. 2023 Jul 11;12:e86182. doi: 10.7554/eLife.86182 (PMC10435230; doi:10.7554/eLife.86182)
Supplement: Supplementary file 2. — Note that there are two transcript IDs for fas6 (compare to Figure 2—figure supplement 2). [file elife-86182-supp2.docx]

Supplementary File 2. Gene names, gene IDs and transcript IDs of all *fas* genes and the housekeeping gene (*elongation factor 1a*) from the present study. Note that there are two transcript IDs for *fas6* (compare to **Figure 2-figure supplement 2**).

| **Gene name** | **Gene ID** |  | **Transcript ID** |
| --- | --- | --- | --- |
| *fas5* | LOC100678737 |  | XM_008209455.4 |
| *fas6* | LOC100124027 |  | XM_031931029.2 |
|  |  |  | XM_008209237.4 |
| *fas1* | LOC100121447 |  | XM_003423866.5 |
| *fas2* | LOC100122099 |  | XM_001605650.6 |
| *fas3* | LOC100122083 |  | XM_008205679.4 |
| *fas4* | LOC100119597 |  | XM_008214022.4 |
| *elongation factor 1a* | LOC100114226 |  | XM_032596271.1 |
