# Supplementary material for: Decoding the genetic and chemical basis of sexual attractiveness in parasitic wasps
Source: eLife. 2023 Jul 11;12:e86182. doi: 10.7554/eLife.86182 (PMC10435230; doi:10.7554/eLife.86182)
Supplement: Supplementary file 3. — Indicated are retention indices (RI), CHC compound identifications or possible configurations in case of ambiguities, their mean absolute (ng) amounts with their respective absolute standard deviations (sd) as well as their respective relative amounts (in %) compared between wild type (WT, N = 14), control knockdown (GFP RNAi, N = 15), and fas5 knockdown (fas5 RNAi, N = 15) female wasps. To compare the absolute quantities (ng) of 54 single CHC compounds among the different treatments, we employed a sequential Benjamini–Hochberg corrected Mann–Whitney U-test between each pair of treatments: fas5 vs. GFP, fas5 vs. WT, and GFP vs. WT. Significant effects in fas5 knockdown (KD) females are indicated by up- (white) and downwards (black) arrows, corresponding to either up- or downregulation of the absolute compound quantities, respectively. Where compound identifications were ambiguous due to multiple possible methyl-branch positions based on the detected ion pairs, all possible compound configurations are given. [file elife-86182-supp3.docx]

Supplementary File 3. **Comparison of absolute quantities and relative abundances of single CHC compounds between differentially treated female wasps.** Indicated are retention indices (RI), CHC compound identifications or possible configurations in case of ambiguities, their mean absolute (ng) amounts with their respective absolute standard deviations (sd) as well as their respective relative amounts (in %) compared between wildtype (WT, N=14), control knockdown (GFP RNAi, N=15) and *fas5* knockdown (*fas5* RNAi, N=15) female wasps. To compare the absolute quantities (ng) of 54 single CHC compounds among the different treatments, we employed a sequential Benjamin-Hochberg corrected Mann-Whitney U-test between each pair of treatments: *fas5* vs GFP, *fas5* vs WT, and GFP vs WT.. Significant effects in *fas5* knockdown (KD) females are indicated by up- (white) and downwards (black) arrows, corresponding to either up- or down-regulation of the absolute compound quantities, respectively. Where compound identifications were ambiguous due to multiple possible methyl branch positions which could be interpreted based on the detected ion pairs, all possible compound configurations are given.

| RI | Compound IDs / configurations | WT female | | GFP RNAi female | | *Fas5* RNAi female | |  |
| --- | --- | --- | --- | --- | --- | --- | --- | --- |
|  |  | mean±sd (ng) | % | mean±sd (ng) | % | mean±sd (ng) | % | KD effect |
| 2900 | n-C29 | 11.11 ± 2.4 | 4,82 | 9.84 ± 2.46 | 3,95 | 11.75 ± 2.34 | 5,06 |  |
| 2939 | 11-MeC29 | 0.94 ± 0.35 | 0,41 | 1.1 ± 0.37 | 0,44 | 1.34 ± 0.81 | 0,58 |  |
| **2947** | **7-MeC29** | **4.12 ± 1.4** | **1,79** | **5.26 ± 2.24** | **2,11** | **1.42 ± 0.79** | **0,61** | \|  \| \| --- \| |
| 2956 | 5-MeC29 | 0.7 ± 0.22 | 0,30 | 0.8 ± 0.27 | 0,32 | 1.21 ± 0.66 | 0,52 |  |
| 2977 | 3-MeC29 | 0.29 ± 0.13 | 0,12 | 0.32 ± 0.1 | 0,13 | 0.53 ± 0.29 | 0,23 |  |
| 2982 | 5,17-DiMeC29 | 0.47 ± 0.17 | 0,20 | 0.56 ± 0.2 | 0,22 | 0.7 ± 0.32 | 0,30 |  |
| 3000 | n-C30 | 1.24 ± 0.41 | 0,54 | 1.28 ± 0.44 | 0,52 | 1.39 ± 0.45 | 0,60 |  |
| 3015 | 3,7-; 3,11-DiMeC29 | 0.32 ± 0.07 | 0,14 | 0.37 ± 0.08 | 0,15 | 0.41 ± 0.11 | 0,18 |  |
| 3036 | 3,7,11-; 3,7,13-; 3,7,15-; 3,7,17-TriMeC29 | 0.23 ± 0.1 | 0,10 | 0.31 ± 0.14 | 0,13 | 0.33 ± 0.16 | 0,14 |  |
| **3044** | **7-MeC30** | **0.66 ± 0.37** | **0,28** | **0.91 ± 0.48** | **0,37** | **0.03 ± 0.02** | **0,01** |  |
| **3054** | **5-MeC30** | **0.04 ± 0.02** | **0,02** | 0.05 ± 0.02 | 0,02 | **0.11 ± 0.06** | **0,05** |  |
| **3061** | **4-MeC30** | **0.05 ± 0.02** | **0,02** | **0.05 ± 0.01** | **0,02** | **0.18 ± 0.05** | **0,08** |  |
| **3087** | **9-C31ene** | **0.62 ± 0.34** | **0,27** | **0.83 ± 0.47** | **0,33** | **2.77 ± 1.44** | **1,19** |  |
| **3096** | **7-C31ene** | **0.41 ± 0.15** | **0,18** | **0.39 ± 0.16** | **0,16** | **1.12 ± 0.38** | **0,48** |  |
| 3100 | n-C31 | 21.35 ± 6.47 | 9,26 | 22.93 ± 7.17 | 9,20 | 25 ± 8.76 | 10,77 |  |
| **3134** | **9-; 11-; 13-; 15-MeC31** | **11.54 ± 4.65** | **5,01** | **14.52 ± 6.83** | **5,83** | **6.88 ± 2.97** | **2,97** |  |
| **3140** | **7-MeC31** | **15 ± 6.4** | **6,51** | **19.23 ± 8.86** | **7,72** | **1.38 ± 0.61** | **0,59** |  |
| **3150** | **5-MeC31** | **4.88 ± 1.19** | **2,12** | **4.9 ± 1.53** | **1,97** | **11.52 ± 5.01** | **4,96** |  |
| **3157** | **11,15-; 11,17-; 11,19-; 13,15-; 13,17-; 13,19-DiMeC31** | **0.42 ± 0.34** | **0,18** | **0.4 ± 0.24** | **0,16** | **0.7 ± 2.43** | **0,30** |  |
| **3173** | **7,11-DiMeC31** | **2.2 ± 0.78** | **0,96** | **2.29 ± 1.06** | **0,92** | **0.51 ± 0.2** | **0,22** |  |
| **3178** | **3-MeC31** | **6.05 ± 1.45** | **2,62** | **5.82 ± 2.22** | **2,34** | **10.21 ± 3.75** | **4,40** |  |
| 3187 | 5,15-; 5,17-; 5,19-DiMeC31 | 0.27 ± 0.1 | 0,12 | 0.26 ± 0.1 | 0,11 | 0.18 ± 0.08 | 0,08 |  |
| **3211** | **3,11-; 3,13-; 3,15-DiMeC31** | **1.27 ± 0.37** | **0,55** | **1.42 ± 0.36** | **0,57** | **6.14 ± 2.1** | **2,64** |  |
| 3217 | 3,7-; 3,9-DiMeC31 | 0.73 ± 0.2 | 0,32 | 0.66 ± 0.18 | 0,27 | 0.93 ± 0.22 | 0,40 |  |
| 3236 | 3,7,9-; 3,7,11-; 3,7,15-TriMeC31 | 2.03 ± 0.64 | 0,88 | 2.35 ± 0.92 | 0,94 | 2.12 ± 0.55 | 0,91 |  |
| **3253** | **6-MeC32** | **0.21 ± 0.08** | **0,09** | **0.21 ± 0.1** | **0,08** | **0.04 ± 0.02** | **0,02** | \|  \| \| --- \| |
|  |  |  |  |  |  |  |  |  |
| RI | Compound IDs / configurations | WT female | | GFP RNAi female | | *Fas5* RNAi female | |  |
|  |  | mean±sd (ng) | % | mean±sd (ng) | % | mean±sd (ng) | % | KD effect |
| 3263 | 3,7,11,15-TetraMeC31 | 1.9 ± 0.5 | 0,82 | 2.03 ± 0.59 | 0,81 | 2.22 ± 0.46 | 0,95 |  |
| **3285** | **9-C33ene** | **0.31 ± 0.17** | **0,13** | **0.37 ± 0.14** | **0,15** | **1.59 ± 0.68** | **0,68** |  |
| **3293** | **7-C33ene** | **0.76 ± 0.32** | **0,33** | **0.74 ± 0.16** | **0,30** | **3.27 ± 1.2** | **1,41** | \|  \| \| --- \| |
| 3300 | n-C33 | 1.02 ± 0.32 | 0,44 | 1.17 ± 0.35 | 0,47 | 1.32 ± 0.47 | 0,57 |  |
| 3334 | 9-; 11-; 13-; 15-MeC33 | 18.35 ± 5.89 | 7,96 | 22.55 ± 8.89 | 9,05 | 14.08 ± 4.07 | 6,06 |  |
| **3347** | **7-MeC33** | **1.52 ± 0.59** | **0,66** | **1.98 ± 0.72** | **0,79** | **0.1 ± 0.08** | **0,05** |  |
| **3353** | **5-MeC33** | **9.59 ± 3.36** | **4,16** | **9.78 ± 3.83** | **3,92** | **3.83 ± 0.75** | **1,65** |  |
| **3364** | **11,15-; 11,17-; 11,19-; 11,21-; 11,23-; 11,27-; 13,15-; 13,17-; 13,19-; 13,21-; 13,23-; 13,27-DiMeC33** | **10.25 ± 3.96** | **4,44** | **10.8 ± 4.1** | **4,34** | **2.95 ± 0.73** | **1,27** |  |
| **3371** | **7,15-; 7,17-; 7,19-; 7,21-; 23-DiMeC33** | **4.38 ± 1.54** | **1,90** | **4.63 ± 1.9** | **1,86** | **1.09 ± 0.38** | **0,47** |  |
| **3382** | **5,9-; 5,11-; 5,15-; 5,17-; 5,21-; 5,23-DiMeC33** | **7.36 ± 2.85** | **3,19** | **7.64 ± 2.24** | **3,06** | **32.68 ± 11.77** | **14,08** |  |
| **3384** | **11,13,21-; 11,15,21-; 11,17,21-; 11,13,23-; 11,15,23-; 11,17,23-TriMeC33** | **3.55 ± 1.48** | **1,54** | **3 ± 1.07** | **1,20** | **1.03 ± 0.2** | **0,44** |  |
| **3394** | **3,9-; 3,11-; 3,13-; 3,15-; 3,17-DiMeC33** | **3.3 ± 0.84** | **1,43** | **3.2 ± 0.7** | **1,28** | **6.89 ± 2.12** | **2,97** |  |
| 3427 | 5,9,13-; 5,11,13-TriMeC33 | 1.99 ± 0.54 | 0,86 | 2.18 ± 0.74 | 0,87 | 2.31 ± 0.58 | 0,99 |  |
| **3431** | **3,7,11-; 3,7,15-; 3,7,17-; 3,7,19-; 3,7,21-TriMeC33** | 0.89 ± 0.46 | 0,39 | **1.24 ± 0.73** | **0,50** | **0.44 ± 0.38** | **0,19** | \|  \| \| --- \| |
| 3458 | 3,7,11,15-TetraMeC33 | 3.94 ± 0.96 | 1,71 | 4.03 ± 1.16 | 1,62 | 3.3 ± 0.6 | 1,42 |  |
| **3477** | **5,13-; 5,15-; 5,17-; 5,19-DiMeC34** | **0.74 ± 0.23** | **0,32** | **0.71 ± 0.16** | **0,28** | **1.44 ± 0.39** | **0,62** |  |
| 3486 | 4,10-; 4,16-; 4,18-DiMeC34 | 0.18 ± 0.15 | 0,08 | 0.11 ± 0.12 | 0,04 | 0.2 ± 0.19 | 0,09 |  |
| 3531 | 11-; 13-; 15-; 17-MeC35 | 9.74 ± 2.84 | 4,22 | 12.15 ± 4.63 | 4,88 | 7 ± 1.66 | 3,01 |  |
| **3557** | **11,17-; 13,17-; 11,19-; 13,19-; 11,21-; 13,21-; 11,23-; 13,23-DiMeC35** | **26.66 ± 8.46** | **11,56** | **27.57 ± 10.24** | **11,06** | **8.36 ± 1.98** | **3,60** |  |
| **3568** | **7,19-; 7,21-; 7,23-DiMeC35** | **4.88 ± 1.4** | **2,12** | **4.48 ± 1.91** | **1,80** | **0.56 ± 0.33** | **0,24** |  |
|  |  |  |  |  |  |  |  |  |
| RI | Compound IDs / configurations | WT female | | GFP RNAi female | | *Fas5* RNAi female | |  |
|  |  | mean±sd (ng) | % | mean±sd (ng) | % | mean±sd (ng) | % | KD effect |
| **3579** | **5,15-; 5,17-; 5,21-; 5,23-; 5,25-DiMeC35** | **9.25 ± 3.04** | **4,01** | **8.9 ± 3.11** | **3,57** | **29.75 ± 10.82** | **12,81** |  |
| **3591** | **3,17-; 3,19-; 3,21-; 3,23-; 3,25-DiMeC35** | **1.13 ± 0.48** | **0,49** | 1.02 ± 0.49 | 0,41 | **0.56 ± 0.24** | **0,24** |  |
| **3599** | **5,9,13-; 5,9,15-; 5,9,17-; 5,9,19-; 5,9,21-TriMeC35** | **1.66 ± 0.56** | **0,72** | **1.46 ± 0.42** | **0,58** | **3.84 ± 1.37** | **1,65** |  |
| **3648** | **3,13,19-; 3,13,23-; 3,13,25-; 3,17,19-; 3,17,23-; 3,17,25-; 3,19,23-; 3,19,25-; 3,23,25-TriMeC35** | **2 ± 0.62** | **0,87** | **1.98 ± 0.71** | **0,79** | **1 ± 0.23** | **0,43** | \|  \| \| --- \| |
| 3722 | 13-; 15-; 17-; 19-MeC37 | 1.81 ± 0.59 | 0,79 | 2.31 ± 0.94 | 0,93 | 1.52 ± 0.44 | 0,65 |  |
| **3744** | **11,17-; 11,19-; 11,21-; 11,23-; 13,17-; 13,19-; 13,21-; 13,23-DiMeC37** | **11.62 ± 3.61** | **5,04** | **11.79 ± 4.74** | **4,73** | **3.03 ± 0.88** | **1,30** |  |
| **3757** | **7,21-; 7,23-; 7,25-DiMeC37** | **1.55 ± 0.54** | **0,67** | **1.38 ± 0.63** | **0,56** | **0.16 ± 0.09** | **0,07** |  |
| **3767** | **5,15-; 5,17-; 5,23-; 5,25-; 5,27-DiMeC37** | **3.11 ± 1.01** | **1,35** | **2.96 ± 0.92** | **1,19** | **8.77 ± 3.22** | **3,78** |  |
|  |  |  |  |  |  |  |  |  |
